# Prevalence of Eating Disorder Risk and Body Image Dissatisfaction among ROTC Cadets

**DOI:** 10.3390/ijerph17218137

**Published:** 2020-11-04

**Authors:** Allison Smith, Dawn Emerson, Zachary Winkelmann, Devin Potter, Toni Torres-McGehee

**Affiliations:** 1 Department of Exercise Science, University of South Carolina, Columbia, SC 29208, USA; abs16@email.sc.edu (A.S.); winkelz@mailbox.sc.edu (Z.W.); 2 Department of Health, Sport, and Exercise Sciences, University of Kansas, Lawrence, KS 66045, USA; dawn.emerson@ku.edu; 3 School of Medicine, University of South Carolina, Columbia, SC 29209, USA; Devin.Potter@uscmed.sc.edu

**Keywords:** tactical athlete, behavioral health, body dysmorphia, body image, body image dissatisfaction, military

## Abstract

Injury risk is multifactorial including non-modifiable and modifiable factors such as nutrition and mental health. The purpose of this study was to estimate eating disorder risk and body image (BI) dissatisfaction among Reserve Officers Training Corps (ROTC) cadets. A total of 102 (male: *n* = 75, female: *n* = 27; age: 20 ± 2 years) ROTC cadets self-reported height, current and ideal weight, and completed the Eating Attitudes Test-26 (EAT-26) and self-perceived BI current and perceived sex-specific figural stimuli. The overall eating disorder risk for ROTC cadets was 32.4%. No significant differences were found when comparing sex, ethnicity, or military branch. Overall risk of pathogenic behaviors included 11.8% who reported binge eating; 8.8% who used laxatives, diuretics, or diet pills; 8.8% who exercised for >60 min to control their weight; and 8.8% who lost 9.1 kg or more within the last 6 months. We identified significant interactions (*p* ≤ 0.01) between sex of the solider, overall perceptions of male and female soldiers, and BI self-perceptions. The ROTC cadets in this study displayed eating disorder risk and BI dissatisfaction, which is concerning for tactical readiness, long-term behavioral health issues, and injury from pathogenic behaviors. Education and quality healthcare are necessary to mitigate the increased risk of eating and BI dissatisfaction within this population.

## 1. Introduction

The Diagnostic and Statistical Manual of Mental Disorders, 5th edition (DSM-5) [1] has categorized eight conditions related to feeding and eating disorder (FED), as well as body dysmorphic disorder, as diagnosable mental health disorders. These mental health disorders include pica, rumination disorder, avoidant/restrictive food intake disorder, anorexia nervosa (AN), bulimia nervosa (BN), binge-eating disorder, other specified FEDs based on frequency or duration, and other unspecified FEDs based on clinically significant distress but not meeting the diagnostic criteria. When a person does not meet the frequency and duration criteria for the eight previously mentioned conditions, but still experiences behaviors and attitudes of maladaptive feeding and weight management, that is considered term disordered eating [2]. The onset of an FED is a complex process that can be triggered for several reasons including weight and body image (BI) dissatisfaction.

When examining individual eating disorders, incidence rates over a 7-year period in the United States Military Academy for females and males respectively were 0.020% vs. 0.000% for anorexia, 0.170% vs. 0.003% for bulimia, and 0.170% vs. 0.020% for eating disorders not otherwise specified (EDNPS) [3]. Additionally, when specifically looking at females in the army, 33.6% who were from the general population on active duty met the criteria for at-risk abnormal eating behaviors; [4] and 20% of female ROTC cadets reported they were at risk for an eating disorder [5]. There has been little research focused on the male military population. Accurately identifying potential FED risk factors and behaviors in physically active males and females (e.g., military) is of high importance [6,7] and the DSM-5′s new disorder categories allow for more precise inclusion and treatment strategies.

The Female and Male Triad Coalition [8] as well as the International Olympic Committee [9] both recognize that FEDs are a precursor to low energy availability, a component of the female and male athlete triad. The female and male athlete triad is a phenomenon that is described as an interrelation of low energy availability with or without an eating disorder, endocrine system dysfunction, and low bone mineral density [8]. Triad research has reported eating disorders as a catalyst for the other components of the Triad, which can lead to long term health consequences which include an increased risk for musculoskeletal injuries, increased risk for sickness, reduced healing time, and infertility [6,8]. There are a variety of tools available to help identify eating disorder which include the Eating Attitudes Test (EAT-26), the Eating Disorder Inventory (EDI-3), and the Eating Disorder Examination Questionnaire (EDE-Q) [10,11,12]. All of the tools can be completed in a short period of time and have frequently been documented as resources to assess eating disorder behaviors [13]. The presence and prevalence of FEDs is evident in the military, but the theoretical framework for behavior influence is often rooted in the training and development of an individual [14].

For the military, the preparation is often during basic training or in a co-cooperative educational program through colleges and universities called the United States Reserve Officer Training Corps (ROTC). The ROTC program allows citizens to attend college while training to enter the United States military as a commissioned officer. Participants in the ROTC program experience the demands of a traditional college student, with the additional stressors of military classes and daily physical training programs [15,16]. With the multiple tasks ROTC cadets endure (e.g., college curriculum, military training, volunteer commitments, etc.), there is an increased pressure to stay fit and active to perform the physical requirements of every military branch. Cadets and officers at every level of the military are required to maintain a specific body mass index (BMI) and physical fitness test benchmarks [4,17], which are conducted biannually. Physical training requirements for the respective branches are used to determine combat readiness and maintain military appearance [17]. If BMI measurements exceed the set standard, one could face being assigned a weight management and fitness program or possible dismissal from the military [18].

Cadets in ROTC programs experience pressures similar to those experienced by a traditional military cadet in basic combat training to maintain weight and pass physical training requirements; however, the addition of being a student enrolled in classes simultaneously may be an additional risk factor for FEDs and disordered eating behaviors. The prevalence of these risk factors for ROTC cadets is like that of student-athletes [8,19]. However, it is important to note a critical difference between the ROTC cadets and student-athletes, which is that cadets do not have immediate access to high quality healthcare in the form of an athletic trainer. The military, and specifically the ROTC, are an emerging setting within the field of athletic training; therefore, there is little research on eating disorder and BI dissatisfaction risk for ROTC male and female cadets. Exploring the presence and prevalence of eating disorder risk and BI dissatisfaction is critical prior to cadets entering the military as commissioned officers due to the additional mental stress of military jobs (i.e., combat training, facing possible injury or death). The primary purpose of our study was to estimate eating disorder risk differences between sex, ethnicity, and branch among ROTC cadets. A secondary purpose was to examine perceived and desired BI of oneself and perceived and desired BI of a soldier (i.e., what one thinks another soldier should look like).

The findings from this study will provide insight into ROTC cadets’ nutritional habits and eating behaviors prior to their enlistment in the armed forces. Additionally, these findings will help individuals who work with tactical athletes (military, law enforcement, etc.) better understand the potential for mental health disorders and the modifiable risk factors of nutrition and mental health that often impact tactical readiness and have long-term influence on behavioral and physical health for the United States military.

## 2. Materials and Methods

We used a cross sectional survey study design. A convenience sample of ROTC cadets was recruited from three Southeastern universities with ROTC programs (i.e., Army, Navy, Marines, Air Force). To be included in the study, participants had to be enrolled in a university ROTC program as cadets and were between the ages of 18 and 35. No exclusion criteria were used for participation in the study. All subjects gave their informed consent for inclusion before they participated in the study. The study was conducted in accordance with the Declaration of Helsinki, and the protocol was approved by the Ethics Committee of the University of South Carolina (Project identification code Pro00032759).

### 2.1. Instrumentation

#### 2.1.1. Demographic Assessment

The demographic assessment was used to identify sex, age, ethnicity, military branch, and self-reported height and weight.

#### 2.1.2. Eating Attitudes Test (EAT-26)

The EAT-26 was used to determine individuals at risk for FEDs by using standardized measures of eating attitudes and behaviors. Questions from the EAT-26 form three subscales which include attitudes relative to dieting, bulimia, and food preoccupation/oral control. The EAT-26 utilizes 26 questions related to eating attitudes and 5 additional questions related to identifying pathogenic behaviors such as purging, binge eating, use of weight loss supplements, and use of laxatives. To be considered at risk, participants scored greater than 20 and/or they met the criteria for pathogenic behaviors; and those who scored below 20 with no pathogenic behavior risk were deemed not at risk for eating disorder behaviors [10]. The EAT-26 questionnaire has been validated and used in previous studies with military cadets [20], has reliability of 0.90 [10], and the reliability for this study was 0.79.

#### 2.1.3. Sex-Specific Figural Stimuli

**Figure 1:**
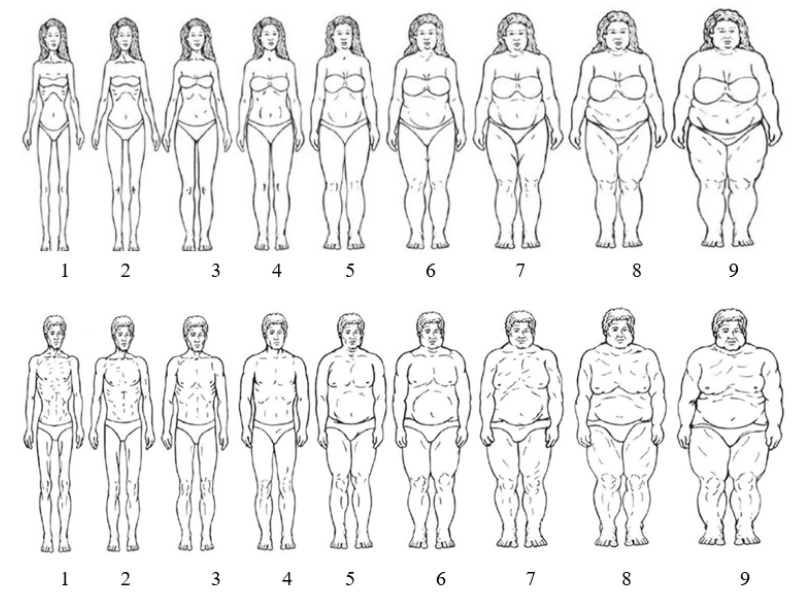
Sex-Specific Figural Stimuli Silhouettes. Female BMI anchors: 1= 17.8, 2= 18.8, 3= 20.3, 4= 22.6, 5= 26.4, 6 = 31.3, 7 = 36.7, 8= 40.8, 9= 44.1. Male BMI anchors: 1= 18.8, 2= 20.2, 3= 21.4, 4= 22.9, 5= 25.4, 6= 28.2, 7 = 33.1, 8= 35.8, 9= 49.4. [21].

The sex-specific figural stimuli is a Likert-style instrument used to assess perceived and desired BI [21] (Figure 1). The tool uses nine pictorial silhouette images for each sex which depict body shapes denoted by a letter. Silhouettes have anchors related to body mass and are denoted in Figure 1 [21]. Participants were asked to report which silhouette best represented their current self-perceived BI and their self-identified desired BI. Next, each participant was asked similar questions relative to their current perceived BI of the male and female cadet and their expected desired BI of the male and female cadet. Polyserial correlations between the logarithm of BMI and the figural stimuli have been well established for current self-perceived BI and self-identified desired BI [21]. In addition, the tool has high reliability for females’ perceived BI (r = 0.85) and desired BI (r = 0.82), as well as males’ current BI (r = 0.79) and ideal BI (r = 0.83) [21].

### 2.2. Procedures

The research team, as well as ROTC leadership, conducted briefings to share study details and recruit the cadets into the study. These briefings were held at the end of the ROTC physical training sessions. The cadets that were interested in participating provided the research team their email address, and then participants were emailed a link for the survey which they could complete without the presence of ROTC leadership. The web-based online survey (SurveyMonkey, San Mateo, CA) included a consent letter and the demographic assessment survey, followed by two validated instruments assessing eating disorder risk and BI. The survey was open for six weeks and was redistributed to all participants 10 days following the initial briefing, and reminders were sent out every 10 days until the window for data collection closed.

### 2.3. Data Analysis

Data were collected and exported from the web-based survey platform to a commercially available statistical software (SPSS Inc., Version 26, Armonk, NY, USA) for all analyses with an alpha level set at *p* < 0.05. We used G*Power software (3.1.9.4) (Heinrich Heine Universitat Dusseldorf, Düsseldorf, Germany) [22] to calculate power. Using an alpha of 0.05 and a moderate effect size (0.3), our power calculation indicated we needed a sample of 88 total participants, with estimated power being 0.90. Basic descriptive statistics including frequencies, means and standard deviations, and calculated differences between self-reported current and ideal weight were used for examining demographic information, the EAT-26 total score and subscales, and the sex-specific figural stimuli score. An independent samples t-test was used to compare EAT-26 totals, raw scores for subscales (dieting, bulimia, and oral control) scores, and sex of the participant. A one-way ANOVA was used to compare the EAT-26 total and subscales (dieting, bulimia, and oral control) scores with the participant’s military branch. Multiple Chi-square analyses were used to examine the proportion of participants classified as “at risk” for eating disorder with the participants’ sex, ethnicity, and military branch. A 2’s (sex: female, male) x 2’s (perception: self-perceived BI, self-identified desired BI) repeated measures ANOVA examined differences between BI self-perceptions and sex. Finally, a 2’s (sex: female, male) x 2’s (sex of soldier: female, male) x 2’s (perceptions: perceived BI of the ideal soldier, desired BI of the ideal soldier) repeated measures ANOVA examined differences between the sex of cadet, the sex of soldier, and the body image perceptions by the cadet of the soldier.

## 3. Results

### 3.1. Participants Characteristics

The participants were on average 20 ± 2 years old and majority were in the Army (*n* = 38, 37.3%) or Navy (*n* = 20, 19.6%) with fewer participants from the Marines (*n* = 9, 8.8%) and the Air Force (*n* = 7, 6.9%) and those choosing to not disclose (*n* = 28, 27.5%). The self-reported physical measurements of the participants are displayed by sex in Table 1.

### 3.2. Prevalence of Eating Disorder Risk and Pathogenic Behaviors

Overall, 32.4% (*n* = 33/102) of participants were classified as being at risk for developing an eating disorder. There were no significant differences in the proportion of those “at risk” for ED between sex, military branch, and ethnicity. However, higher rates were seen among males, whites, and Army cadets. Upon further evaluation of demographic variables (e.g., height, weight, etc.), no significant differences were found for sex (χ^2^ = 2.5, *p* = 0.18), ethnicity (χ^2^ = 3.9, *p* = 0.27), or military branch (χ^2^ = 2.3, *p* = 0.51). Eating disorder risk results are displayed in Table 2.

Of the 33 participants identified as at risk, the type of risk (i.e., EAT-26 risk, pathogenic behavior risk, or a combination) was variable, which is demonstrated in Table 3. Pathogenic behavior risk was highest among males (26.7%, *n* = 20), Asians (66.7%, *n* = 2), and Army cadets (31.6%, *n* = 12). When examining pathogenic behaviors, significant differences were found between sex and use of diet pills, diuretics, or laxatives to lose weight (females: 25.9% (*n* = 7); males: 2.7% (*n* = 2); χ^2^ = 13.35, *p* < 0.001) which can be found in Table 4.

### 3.3. Body Image

**Figure 2:**
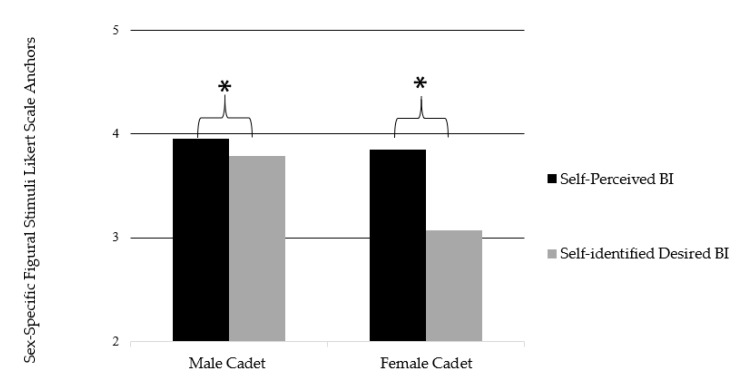
Body Image Scores using Sex-Specific Figural Stimuli Likert Scale Anchors [21]. * Denotes significant differences at the *p* ≤ 0.01 level.

**Figure 3:**
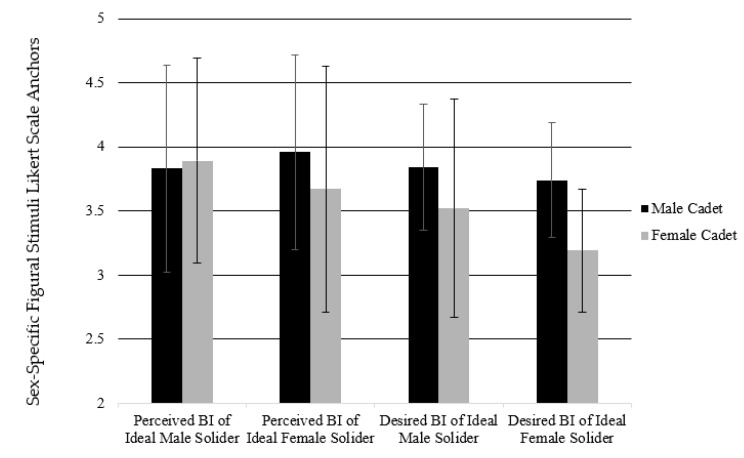
Body Image Perceptions reported by cadets on soldiers. Scores using sex-specific figural stimuli Likert Scale Anchors [21].

The repeated measures ANOVA revealed a main effect between sex and BI self-perceptions (F1,100 = 22951.19, *p* = 0.01, η^2^ = 0.996). Significant interactions were found with perception (F1,100 = 32.28, *p* = 0.01, η^2^ = 0.244) and perception and sex (F1,100 = 12.57, *p* = 0.01, η^2^ = 0.112). When exploring female and male cadet BI perceptions of male and female soldiers, a significant main effect was found between cadet perceptions (perceived BI, desired BI) in both male and female soldier (F1,100 = 48556.96, *p* = 0.01, η^2^ = 0.998). Significant interactions were found with perceptions (F1,100 = 12.82, *p* = 0.01, η^2^ = 0.114), perceptions and sex (F1,100 = 5.82, *p* = 0.018, η^2^ = 0.055), ideal sex (F1,100 = 22.75, *p* = 0.01, η^2^ = 0.185), ideal sex and sex (F1,100 = 4.30, *p* = 0.41, η^2^ = 0.041), and perception and ideal sex (F1,100 = 7.6, *p* = 0.01, η^2^ = 0.071). All BI perception data can be found in Table 5 and Figure 2 and Figure 3.

## 4. Discussion

### 4.1. Eating Disorder Risk

The purpose of this study was to estimate eating disorder risk and determine differences between sex, ethnicity, and branch among ROTC cadets while additionally examining BI dissatisfaction among sex and BI perceptions of a soldier. The overall estimate for eating disorder risk for ROTC cadets was 32.4%, which is consistent with traditional athletes [23] and previously published literature surrounding military personnel which has been reported as ranging from 21%–45.5% [24,25].

When examining by sex, males and females reported at risk for eating disorder at rates of 28.0% and 44.4% respectively. This finding is inconsistent with reported rates from a systematic review of military and veteran populations which states male prevalence rates of eating disorders ranging between 0.1%–3.9% and females ranging from 2.8%–8% [3,26]. The high rates within this population are concerning due to the age of typical ROTC cadets being younger than that of traditional military personnel. Numerous studies have found that service members within the younger age group experienced elevated rates of eating disorder pathologies [17,27,28,29]. While this study has significantly larger prevalence rates when compared to previous literature, it is important to note all the studies included in the systematic review utilized diagnosis criteria from the DSM-IV. Since the implementation of the DSM-5, prevalence rates have changed due to the addition of several diagnosable disorders and more detailed criteria. For example, diagnosed cases of AN increased from 29.6% to 33.5% and cases of BN increased from 22.7% to 24.7% [30]. Using the outdated DSM-IV criteria, a third of patients who were diagnosed were placed in the EDNOS category, whereas the DSM-5 now allows patients to receive specific treatment which may help with increased overall health.

To date, there have been no studies that examine eating disorder risk in the military regarding ethnicity. Within our study sample, Black cadets were found to have the highest “at risk” percentage (54.5%); however, White, and Asian participants reported at risk percentages close to a third of individual samples. This finding is interesting due to the Black sample self-reporting the lowest current weight among all ethnicities. In contrast to our findings, Akan et al., [31] found Black females had the largest reported BMI within a sample of college females, and the White participants reported the highest levels of eating disorder behaviors and attitudes which was attributed to low self-esteem and high public self-consciousness. These findings support the need for a transition within the military to a more personalized healthcare approach. Without proper education and change within all military branches, ROTC cadets and current soldiers who are commissioned into a military branch could continue to work towards ideals of physical appearances that are not achievable. This unachievable standard continues to put these cadets and soldiers at risk for long term health complications which could affect their readiness to perform within a military setting (i.e., combat, physical training, etc.).

### 4.2. Pathogenic Behaviors

When examining the overall eating disorder risk and pathogenic behaviors with the EAT-26, it is important to determine the specific risk category that the individual falls within (i.e., EAT-26 risk, pathogenic behavior risk, or a combination). For this sample, the highest reported frequency was found with pathogenic behaviors by themselves and the most reported behavior was binge eating. ROTC cadets reported higher percentages in more behavior categories than any previously reported population, such as college athletes [23]. All the military branches have specific and strict physical requirements that could lead to the potential for utilization of pathogenic behaviors. Research has noted that 27% of military recruits report crash dieting and even more (67%) report implementing a diet within two months of physical fitness testing [32]. While all the pathogenic behaviors included in this study are precursors to diagnosable mental health disorders, there are also the physical implications involved in repetitive and long-term use. Behaviors such as self-induced vomiting, use of laxatives, dietetics, and diet pills can lead to a host of gastrointestinal issues [33]. These gastrointestinal issues can also lead to immune system suppression which leads to the risk of frequent sickness [6]. Additionally, these behaviors are commonly linked with bipolar disorder, depression, and anxiety disorders [34]. If not addressed early on, all the potential results of continued utilization of pathogenic behaviors could lead to decreased physical and mental abilities of cadets and soldiers and ultimate mission failures.

### 4.3. Body Image

Our study is unique in the fact that it is the first to examine self-perceptions related to BI of male and female ROTC cadets while also examining ideal perceptions of what a combat soldier should look like. Male and female cadets displayed a significant difference when comparing their perceived BI and their reported desired BI, and both sexes desired to be smaller in body shape and size than their current perception; however, females had a larger discrepancy (males: 23.13 vs. 22.59; females: 22.88 vs. 20.56). A theory for why these individuals would desire to be smaller is the military ideal that those who have normal BMI have less likelihood of injury or discharge from training [35]. There has been inconsistent data on the relationship of BMI and risk of injury [36], however those at the extreme high (BMI > 33) are at the greatest risk of medical discharge [37]. The US military physical fitness assessments focus on strength (timed pushups and sit ups) as well as endurance (timed 2-mile run) which stresses multiple musculoskeletal points throughout the body (i.e., wrist joints during pushups, back, hip, knee, and ankle joints during the 2-mile run). Those individuals with large BMI have a higher rate of orthopedic injuries during these physical fitness assessments [38,39]. Our results related to females are consistent with previous body image on female college athletes [23]; however, the male results are inconsistent. Generally, most college females report having low levels of body image satisfaction coupled with the desire to be significantly smaller in body size [40]. Male athletes report wanting to ideally be larger and more muscular in shape [41]. However, in the military these norms do not always hold true.

When examining the perceptions of a soldier from the cadet’s perspective, we found that male cadets reported an increase in size of the male soldier (perceived smaller than ideal) while female cadets reported a decrease (perceived larger than ideal) in size. When focused on the female soldier, both male and female cadets reported a decrease in size from perceived to ideal size, however the female cadets reported a much larger discrepancy between current perceived and ideal size of the soldier. This finding is concerning due to previous literature within the military reporting that females within ROTC and formal military settings are already at risk for abnormal eating habits and eating disorder risk [5,17]. Additionally, these results suggest potential BI dissatisfaction, a preoccupation with one’s own body [1], for those females who continue on through the ROTC program into the formal military. With most ROTC cadets being of traditional college age (18–23) and then continuing to military service, there is a potential for long term disability. Another major concern is that BI dissatisfaction is comorbid with depressive disorder, while social anxiety disorder, obsessive compulsive disorder, and substance abuse are also possible [1]. These comorbidities all can provide negative effects on ones’ long-term mental and physical health as well as military service.

While this study was one of the first to examine eating disorder risk and BI dissatisfaction within the ROTC population, the following limitations should be considered. First, this study utilized self-reported measures for both ED risk and BI risk; therefore, utilizing self-reported measures may come with discomfort in answering questions honestly. Additionally, the EAT-26 is a screening tool and not a diagnostic tool, because of this we cannot conclude that those cadets that are classified as at risk have an eating disorder. We utilized the sex-specific figural stimuli silhouettes to assess BI. There are other instruments that can be used to assess BI and self-perceptions (Eating disorder inventory 3, etc.); however, those tools are costly and can be time consuming. Another limitation would be the small number of Black and Asian participants within the sample (*n* = 14, 13.7% collectively) and the disproportion of sex. However, when compared to the active duty military population, Black and Asian ethnicities were reported to make up 13.9% of the population and the males accounted for 84.1% of the population which is higher than our sample [42]. A final limitation would be that our study was limited to ROTC cadets within the southeastern region of the United States. Future research should expand the scope of eating disorder risk and BI dissatisfaction to ROTC cadets in other regions of the United States as well as other levels of the military. We also suggest exploring the influence of educational sessions by healthcare professionals such as athletic trainers on the presence of eating disorder risk and BI dissatisfaction relative to long-term behavioral changes in cadets and soldiers.

## 5. Conclusions

We found ROTC cadets were at risk for eating disorder and pathogenic behaviors and also experienced BI dissatisfaction. Reserve Officer Training Corps cadets must balance the demand of physical training sessions and traditional academic course work with the addition of military-focused classes, much like a student athlete must find harmony between athletic responsibility and academic coursework. These results for overall eating disorder risk within the ROTC cadet population highlight the need for education by healthcare professionals of individuals at all levels of military service in relation to healthy eating habits and consequences such as the Triad. However, cadets are often only provided with access to general practice physicians through student health services on campus whereas athletes have access to health care professionals such as athletic trainers, sports medicine physicians, and dietitians. By providing ROTC cadets and other military settings with athletic trainers, these mental health problems, in addition to orthopedic injury and illness, can be screened for, prevented, detected, and treated during real time. Identification of these behaviors allows for modification in training and additional educational opportunities which can enhance the overall health and wellbeing of ROTC cadets as they transition into full-time military positions. Screening cadets for predisposing conditions (i.e., disordered eating and components of the Triad) should only be the first level of care. The findings of our study suggest that there is an increased need for a multidisciplinary team (athletic trainers, physicians, registered dieticians, nutritionists, social workers, etc.) who can treat the ROTC cadet population in a holistic manner. This suggested approach could help decrease the risk of suffering an injury or illness that leads to discharge from the military.

## Figures and Tables

**Table 1:** Self-reported physical measurements by demographic variable reported in mean ± standard deviation.

Demographic Variable	Males (*n* = 75)	Females (*n* = 27)
**Self-Reported Height (cm)**	178.6 ± 7.4	164.9 ± 7.8
**Self- Reported Weight (kg)**		
Current Weight	78.1 ± 9.5	63.8 ± 10.2
Ideal Weight	79.2 ± 8.6	60.8 ± 6.9
Highest Weight	83.5 ± 13.4	65.9 ± 10.9
Lowest Weight	72.1 ± 9.6	57.7 ± 9.4
Calculated Difference *	−1.1 ± 6.8	2.8 ± 6.5

* The calculated difference is the participants’ current weight minus their ideal weight in kilograms.

**Table 2:** Proportion of Reserve Officer Training Corps (ROTC) cadets classified as at risk for eating disorders (reported in percent and sample size).

Demographic Variable	Proportion Classified as At Risk in Total Sample	Proportion Classified as At Risk within Group	*p*-Value *
**All ROTC Cadets (*n* = 102)**	32.4% (33/102)		
**Sex**			0.18
Male (*n* = 75)	63.6% (21/33)	28.0% (21/75)	
Female (*n* = 27)	36.4% (12/33)	44.4% (12/27)	
**Race**			0.27
White (*n* = 79)	69.7% (23/33)	29.1% (23/79)	
Black (*n* = 11)	18.2% (6/33)	54.5% (6/11)	
Asian (*n* = 3)	3.0% (1/33)	33.3% (1/3)	
Hispanic (*n* = 7)	3.0% (1/33)	14.3% (1/7)	
Did Not Disclose (*n* = 2)	6.1% (2/33)	100% (2/2)	
**ROTC Branch**			0.51
Army (*n* = 53)	57.6% (19/33)	36.5% (19/52)	
Marines (*n* = 9)	15.2% (5/33)	41.7% (5/12)	
Navy (*n* = 20)	15.2% (5/33)	25.0% (5/20)	
Air Force (*n* = 7)	6.1% (2/33)	20.0% (2/10)	
Did Not Disclose (*n* = 28)	6.1% (2/33)	25.0% (2/8)	

* Denotes differences across sex, race, and ROTC branch.

**Table 3:** ROTC cadets’ type of risk for eating disorder by sex, ethnicity, and military branch presented as % (*n*).

Demographic Variable	EAT-26 Risk *	Pathogenic Behavior Risk **	Eat-26 + Pathogenic Behavior Risk	Not at Risk
	% (*n*)	% (*n*)	% (*n*)	% (*n*)
**All ROTC Cadets**	2.0% (2)	25.5 (26)	4.9% (5)	67.6% (69)
**Sex**				
Male (*n* = 75)	2.7% (2)	26.7% (20)	2.7% (2)	68.0% (51)
Female (*n* = 27)	0% (0)	22.2% (6)	11.1% (3)	66.7% (18)
**Ethnicity**				
White (*n* = 79)	2.5% (2)	26.6% (21)	5.1% (4)	65.8% (52)
Black (*n* = 11)	0% (0)	18.2% (2)	0% (0)	81.8% (9)
Asian (*n* = 3)	0% (0)	66.7% (2)	0% (0)	33.3% (1)
Hispanic (*n* = 7)	0% (0)	14.3% (1)	0% (0)	85.7% (6)
Did not disclose (*n* = 2)	0% (0)	0% (0)	50% (1)	50% (1)
**Military Branch**				
Army (*n* = 38)	2.6% (1)	31.6% (12)	10.5% (4)	55.3% (21)
Marines (*n* = 9)	0% (0)	22.2% (2)	0% (0)	77.8% (7)
Navy (*n* = 20)	5.0% (1)	25.0% (5)	0% (0)	70% (14)
Air Force (*n* = 7)	0% (0)	0% (0)	14.3% (1)	85.7% (6)
Did not disclose (*n* = 28)	0% (0)	25.0% (7)	0% (0)	75.0% (21)

* EAT-26 risk refers to risk attributed from responses to 26 attitude questions summing ≥20. ** Pathogenic behavior risk refers to risk attributed from responses to supplemental questions reflecting behaviors of binge eating, self-induced vomiting, use of diet pills, diuretics, and laxative, excessive exercise, and losing 9.1 kg (20 lbs.) in the last 6 months.

**Table 4:** Proportion of ROTC cadets classified as at risk for specific pathogenic behaviors (percent and sample size).

Demographic Variable	Binge Eating	Vomiting	Diet Pills, Diuretics, and Laxatives	Over Exercise	Lost 9.1 kg
**Overall ROTC Cadets at Risk**	11.8% (12)	2.0% (2)	8.8% (9)	8.8% (9)	8.8% (9)
Sex *					
Males (*n* = 75)	10.7% (8)	1.3% (1)	2.7% (2)	9.3% (7)	8.0% (6)
Females (*n* = 27)	14.8% (4)	3.7% (1)	25.9% (7)	7.4% (2)	11.1% (3)
**Race**					
White (*n* = 79)	11.4% (9)	2.5% (2)	5.1% (4)	8.9% (7)	8.9% (7)
Black (*n* = 11)	27.3% (3)	0% (0)	27.3% (3)	9.1% (1)	9.1% (1)
Asian (*n* = 9)	0% (0)	0% (0)	0% (0)	0% (0)	0% (0)
Hispanic (*n* = 20)	0% (0)	0% (0)	14.3% (1)	0% (0)	0% (0)
Did Not Disclose (*n* = 2)	0% (0)	0% (0)	50.0% (1)	50.0% (1)	50.0% (1)
**Branch**					
Army (*n* = 38)	7.9% (3)	0% (0)	13.2% (5)	15.8% (6)	7.9% (3)
Marines (*n* = 9)	33.3% (3)	0% (0)	11.1% (1)	0% (0)	11.1% (1)
Navy (*n* = 20)	15.0% (3)	10.0% (2)	5.0% (1)	10.0% (2)	5.0% (1)
Air Force (*n* = 7)	14.3% (1)	0% (0)	14.3% (1)	0% (0)	14.3% (1)
Did Not Disclose (*n* = 28)	7.1% (2)	0% (0)	3.6% (1)	3.6% (1)	10.7% (3)

* Indicates significant differences identified between sex and diet pills, diuretics, and laxatives (*p* < 0.001).

**Table 5:** Descriptive statistics for ROTC cadets reported self-perceptions, and perceptions of the ideal soldier. Values presented in mean and standard deviation of Body Mass Index. An α = 0.05 was used to determine significance.

Demographic Variable	Male		Females	
	Perceived BI	Desired BI	Perceived BI	Desired BI
Self-Perceptions of Cadet	23.13 ± 1.65	22.59 ± 0.71	22.88 ± 3.54	20.56 ± 1.09
Perceptions of Male Soldiers	22.86 ± 1.56	22.70 ± 0.84	22.98 ± 1.54	22.21 ± 0.94
Perceptions of Female Soldiers	22.86 ± 2.42	22.00 ± 1.03	22.25 ± 1.03	20.75 ± 1.04

**Note:** BMI mean, and standard deviation were obtained from the sex-specific figural stimuli anchors and calculated for the overall sample and for male and female cadets’ self-perceptions and what they perceived as the ideal male and female soldier.
